# Immune and metabolic disturbance as a function of genetic risk and phase of illness in major depression

**DOI:** 10.1016/j.bbih.2025.101144

**Published:** 2025-11-27

**Authors:** David M. Howard, Lachlan Gilchrist, Petroula Proitsi, Elisabeth R. Paul, Markus Heilig, Lars Östman, Robin Kämpe, J. Paul Hamilton

**Affiliations:** a King's College, London, England; b Center for Social and Affective Neuroscience, Department of Biomedical and Clinical Sciences, Linköping University, Sweden; c Department of Biological and Medical Psychology, University of Bergen, Norway

**Keywords:** Major depression, Polygenic risk, Environmental risk, Immunological, Metabolic, Episodic

## Abstract

Immune and metabolic factors are important in the pathophysiology of major depressive disorder (MDD) but we know little about how these factors manifest in relation to the status of depressive illness—from genetic risk for MDD, to a depressive episode, to depression in remission. Using genetic, diagnostic, biometric, and blood-bioassay data from the UK Biobank, we examined measures of pro-inflammatory signaling (C-reactive protein) and metabolic dysfunction (metabolic syndrome symptomatology) in females (N = 37,806) and males (N = 17,946) as a function of polygenic load for MDD (high versus low) interacting with depression status (never depressed, currently depressed, or depression in remission). We examined socioeconomic status (SES) as an exploratory factor in this design. Groups were matched for several confounders using a propensity-matching algorithm (females: n = 6301 per group for N = 37,806; n = 2991 per group for N = 17,946). In females we found increased inflammation and metabolic dysfunction in the higher-versus-lower PRS quartile, in those below-versus-above the median SES, and in those suffering currently from depression relative to their remitted depressed and healthy counterparts. This association remained when considering only non-psychotropic-medicated persons. Nonetheless, we also saw in both male and female samples that measures of immunological and metabolic dysfunction increased with increasing anti-depressant medication load. We discuss these findings in terms of the epidemiological significance of immune and metabolic functioning in depression and their paradoxical relation with antidepressant treatment.

## 1 Introduction

Immunological dysregulation is observed reliably in major depressive disorder (MDD) (Köhler et al., 2017). While immunological characterizations of MDD have garnered much attention (Köhler et al., 2018), the case for metabolic dysfunction in depression, too, has amassed strong and consistent evidence (Pan et al., 2012a; Bot et al., 2020). Noting associations between metabolic and immunological dysregulation, a recent conceptualization melds these characterizations of MDD with aspects of atypical MDD—such as weight gain, hypersomnia, fatigue, and leaden paralysis (Milaneschi et al., 2020).

Most immunological and metabolic inquiry into MDD has used case-control designs comparing currently depressed and matched, healthy-control samples. These designs have yielded useful and replicable insights (Köhler et al., 2017; Milaneschi et al., 2020) and stimulated important additional investigation. Some of the most pressing questions suggested by these findings concern where to place metabolic and immunological abnormalities in the pathophysiological arc of depressive illness. Are the irregularities part of a premorbid biological risk profile? (Chen et al., 2010) Do they emerge only during a depressive episode, potentially as proximate causal factors? Or are they scars from one or more previous depressive episodes (Stange et al., 2017), conferring risk for future episodes of depression? These questions can be addressed by combining appropriate genotyping—comparing groups at low-versus high-polygenic risk for MDD—and phenotyping—comparing never-depressed, currently depressed, and remitted depressed groups.

Combining genetic, phenotypic, immunological, and metabolomic data can also help address questions about biological and behavioral heterogeneity in MDD, with implications for the nosology of major depression. While there is widespread agreement as to the heterogeneity of major depression (Cannon and Keller, 2006; Labonté et al., 2017; Cuthbert and Insel, 2013), there is resultant uncertainty about how to identify and describe the subclasses comprised by this disorder. Importantly, in this context there are demographic and biological links to immune-metabolic dysfunction in MDD that suggest an integrative conceptualization in which demographic, diagnostic, and genetic, and humoral data cohere. The first link is the female predominance in atypical MDD. A recent investigation based on data from over 37,000 participants who endorsed symptoms of MDD in the UK Biobank Mental Health Questionnaire found that the female-to-male ratio for those affected by atypical MDD (∼3.0:1) was significantly elevated relative to the non-atypical subtypes of depression (∼2.2:1) (Brailean et al., 2020). Heritability also relates uniquely with immune-metabolic dysfunction in depression. This was first suggested by observations of significant familial aggregation for atypical MDD but not melancholic depression (Lamers et al., 2016). A more recent mega-analysis of data from the Psychiatric Genomics Consortium (PGC) showed that patients with MDD endorsing atypical features, relative to their non-atypical MDD counterparts, carried a higher load of genetic variants associated with immune-metabolic dysfunction such as elevated body-mass index and higher blood levels of C-reactive protein—CRP, a broad marker of pro-inflammatory signaling (Milaneschi et al., 2017).

In the present investigation, we examine whether genetic risk (low-versus high-polygenic risk for depression) depression status (never-versus currently-versus remitted-depressed) and the interaction of these factors are associated with immunological dysfunction (elevated CRP) and metabolic dysfunction (more criteria met for metabolic syndrome). Based on the sex and genetic findings presented above, we expect to observe greater immunological and metabolic disturbance in depressed women with a high genetic risk for MDD relative to their low-risk counterparts. The depression-status variable is exploratory, and we make no prediction about whether altered immune-metabolic functioning is present to a greater extent in currently versus remitted depressed persons relative to never-disordered individuals. Complementary to genetic risk for immune-metabolic dysregulation in depression, environmental deprivation is an important risk factor for depression (Qi et al., 2022) that should be examined in immune-metabolic terms. Given this, we assess whether adding a socioeconomic factor to our primary model can account for variability in immunological and metabolic functioning and predict, based on recent meta-analytic findings (Muscatell et al., 2020; Blanquet et al., 2019), that lower socioeconomic status will be associated with elevated inflammation and metabolic dysfunction. Further, given that utilization of many anti-depressant drugs is associated with subsequent weight gain (Gafoor et al., 2018; Alonso et al., 2019), we also examine relations between genetic and disease-status variables and metabolic and immunological factors both including and excluding anti-depressant medicated persons. Relatedly, we explore whether the extent of antidepressant use is associated with alterations in immunological and metabolic function.

## 2 Material and methods

### 2.1 Overview

**Fig. 1:**
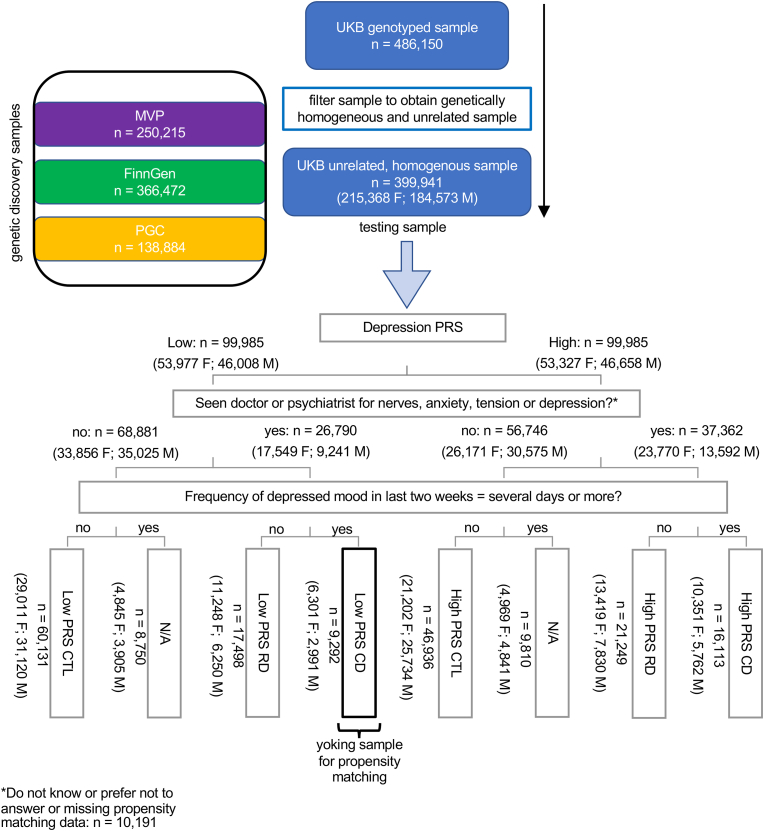
Sorting schematic for genetic discovery and testing and allocating participants from the testing sample to cells of the factorial design permuting Genetic Risk for depression with Depression Status; CD = Currently Depressed; CTL = Control; F = female; M = male; MRI = magnetic resonance imaging; MVP = Million Veterans Program; PGC = Psychiatric Genomics Consortium; PRS = polygenic risk score for depression; RD = Remitted Depressed; UKB = UK Biobank.

Using data from the UK Biobank (UKB; https://www.ukbiobank.ac.uk; accessed under application 49037; see Supplementary Methods for details about the UKB) we examined women and men separately using a two-by-three, between-groups factorial design examining effects of Genetic Risk for depression, Depression Status, and the interaction of these factors in relation to immunological and metabolic function. (See the section *Data analysis*, below, about the rationale for separate analyses for women and men.) To do this, we first calculated genetic risk for depression by meta-analysing the results from genome-wide association studies of FinnGen (Kurki et al., 2023), the Million Veterans Program (MVP) (Levey et al., 2021), and PGC (Wray et al., 2018) datasets. Second, we assigned UKB participants into six groups according to Genetic Risk and Depression Status (see Fig. 1 for sorting schematic): 1) High-PRS Control, 2) High-PRS Currently Depressed, 3) High-PRS Remitted Depressed, 4) Low-PRS Control, 5) Low-PRS Currently Depressed, and 6) Low-PRS Remitted Depressed. In a complementary sensitivity analysis, we examined immunological and metabolic functioning using the same factorial design but excluding anti-depressant medicated persons.

#### 2.1.1 Between-groups factors: Genetic risk

To obtain estimates of genetic risk for depression in the UKB, we used summary statistics from association analyses conducted by FinnGen (Levey et al., 2021) (based on Freeze 9; 43,280 depression cases and 323,192 controls), MVP (Levey et al., 2021) (83,810 cases and 166,405 controls), and PGC (Wray et al., 2018). FinnGen and MVP used electronic-health-record-derived International Classification of Diseases (ICD) codes to define depression cases and controls. PGC meta-analyzed genome-wide association data from 35 cohorts primarily using the ICD or the Diagnostic and Statistical Manual of Mental Disorders to determine case and control status. We used summary statistics that excluded UKB and 23andMe leaving data from 43,204 major depression cases and 95,680 controls. Variants with a minor allele frequency <0.01 or imputation accuracy <0.6 were removed.

To meta-analyze the summary statistics from the discovery samples we used METAL (Willer et al., 2010). Prior to meta-analysis, summary statistics were standardized with MungeSumstats (Murphy et al., 2021), using dbsnp 144 and the BSgenome.Hsapiens.1000genomes.hs37d5 reference genome to obtain missing rsIDs, exclude duplicated or multi-allelic variants and align effect and allele frequencies. MungeSumstats was used to lift FinnGen summary statistics over from GRCh38 to GRCh37. As genomic inflation control in the meta-analysis, we used the regression intercepts calculated using linkage disequilibrium score regression (Bulik-Sullivan, 2015) for FinnGen, MVP, and PGC summary statistics. We used an inverse variance-weighted analysis to weight the effect sizes from each study which provided a total sample size of 170,222 depression cases and 585,277 controls.

To estimate genetic risk for depression in the testing sample we used PRS-CS (Ge et al., 2019) to infer posterior SNP effect sizes using the meta-analyzed summary statistics from the discovery samples. We used the European LD reference panels constructed using the 1000 Genomes Project phase 3 samples available from https://github.com/getian107/PRScs. We used the default parameter values in the gamma-gamma prior (a = 1 and b = 0.5). The global shrinkage parameter (phi) was learnt from the data using a fully Bayesian approach. We used default Markov chain Monte Carlo values (iterations = 1,000, burn in = 500, and thinning factor = 5). This provided posterior SNP effect sizes for 534,123 variants and then Plink v1.9 (Chang et al., 2015) was used to predict the polygenic scores for individuals in the UKB. To obtain an unrelated sample, UKB individuals who were related up to the third degree, based on a kinship coefficient >0.044 (UKB Data-Field 22021), were excluded. Individuals with a genotype call rate <98 % or a sex mismatch and variants with a call rate <98 %, a minor allele frequency <0.01, imputation accuracy <0.7, or a Hardy-Weinberg equilibrium *p*-value <10^−6^ were removed.

#### 2.1.2 Between-groups factors: Depression status

For our two-by-three factorial design, participants from the UKB testing sample were partitioned into six principal categories based on being in the top or bottom quartile of Genetic Risk for depression (High-versus Low-PRS) permuted with Depression Status. We used the ‘broad depression’ phenotype from Howard and colleagues (Howard et al., 2018a) to determine history of depression. Participants were considered depression-history-positive if they answered yes to either “Seen doctor (GP) for nerves, anxiety, tension or depression” or “Seen psychiatrist for nerves, anxiety, tension or depression” (UKB Data-Fields, 2090 and 2100, respectively, acquired at the initial visit). This phenotype has been found to have a high genetic correlation (*r*_*G*_ = 0.86) with clinically assessed major depressive disorder (Howard et al., 2018b). We used the self-reported “Frequency of depressed mood in the last two weeks (“not at all” versus “several days” or more per UKB Data-Field, 2050) to sort depression-history-positive individuals into Currently-versus Remitted-Depressed groups—see sorting schematic presented in Fig. 1. In recent work, we have validated the broad depression phenotype against ICD-based formal clinical assessment available via the UKB data portal. For both female and male samples, individuals in the current and remitted depression groups, relative to controls, were much more likely to have received an ICD-based diagnosis of depression (Χ^2^ = 66.05 and 107.95; and *p* = 4.49e-16 and 2.20e-16, respectively). We have also validated the current depression phenotype against self-reported, recent stressful experiences. Individuals meeting criteria for this phenotype reported significantly more recent stressful events than healthy controls (females: *t* = 7.28, *p* = 6.19e-13; males: *t* = 4.99, *p* = 5.85e-07) and persons meeting criteria for remitted depression (females: *t* = 5.54, *p* = 3.61-e−08; males: *t* = 4.58, *p* = 5.85e-06). Healthy control and remitted depression samples did not differ with regard to recent stressful experiences (Kämpe et al., 2024).

### 2.2 Dependent measures

#### 2.2.1 Metabolic syndrome (MBS) score

National Institutes of Health guidelines define MBS according to the following criteria: 1) large waist (at least 89 cm for men and 102 cm for women; UKB Data-Field 48); 2) high triglyceride level (at least 1.7 mmol per liter; UKB Data-Field 30870); 3) low HDL cholesterol (less than 1.3 mmol per liter; UKB Data-Field 30760); 4) high blood-pressure (at least 130/85 mm Hg; UKB Data-Fields 4079/4080); 5) elevated fasting blood sugar (at least 5.6 mmol per liter; not part of UKB blood biochemistry panel) (Swarup et al., 2024). We calculated an MBS score for each participant by summing the available criteria met (range from 0 to 4) and then dividing by the number of available criteria for each participant. See Supplementary Fig. 1 for a histogram showing the number of participants per the number of available criteria. We calculated a continuous MBS score as opposed to a binary diagnostic index (three or more criteria met) both to reflect the true variability in metabolic dysfunction and to prospectively provide our analytic procedure with additional variance to account for. Moreover, given our intention to characterize metabolic dysfunction at a state-as opposed to a trait-level, our approach did not account for medically managed symptoms.

#### 2.2.2 C-reactive protein

Circulating CRP levels rise in response to inflammatory challenge and are considered a proxy for pro-inflammatory activity (Kragsbjerg et al., 1995; Ohzato et al., 1992). We therefore included CRP (immunoturbidimetric, high-sensitivity analysis using a Beckman Coulter AU5800; UKB Data-Field 30710) as the index of inflammation in our investigation.

### 2.3 Data analysis

#### 2.3.1 Covariates/confounder control

To address any confounding effects of selection bias based on genetic loading for depression and/or depression status we used a propensity score matching approach. First, based on principles of confounder selection (VanderWeele, 2019), we identified ten variables potentially conflated with Genetic Risk and/or Depression Status that could also affect metabolic and immunological variables. These variables were age (UKB Data-Field 21002) (Toth and Tchernof, 2000), smoking (UKB Data-Field 1239, current tobacco smoking recoded so that larger numbers reflect more severe habit) (Levitzky et al., 2008), drinking (UKB Data-Field 1558, current alcohol intake frequency recoded so that larger numbers reflect higher alcohol intake) (Imhof et al., 2001), genotyping array, and the first six principal components of the whole-genome data of the testing sample. In conducting propensity matching, we used the smallest group in the two-by-three design (Low-PRS Currently Depressed) as the “yoking group.” Thus, a subset of each of the other groups was matched, one to one, with subjects from this group. Given that the incidence of depression is about twice as high in women as men, to bolster total sample size we propensity-matched and analyzed data from women and men separately as opposed to yoking the full sample to the smallest group (Low-PRS Currently Depressed men). We used a nearest-neighbor matching algorithm available in the R package MatchIt (Stuart et al., 2011) to match subjects in terms of propensity. This approach yielded six propensity matched groups (females: n = 6,301, N = 37,806; males: n = 2,991, N = 17,946) and was effective at matching samples with respect to potential demographic, health-related, and clinical confounders; see Supplementary Fig. 2 for an example. In Supplementary Table 1, we present main effects and interactions for all potential confounders following propensity score matching.

#### 2.3.2 Ancillary analyses

To investigate associations between Socioeconomic Status (SES) and immunological and metabolic functioning in addition to potential interactions between SES and Genetic Risk and Depression Status factors, we added an SES factor to the design by performing a median split of each cell in the primary two-by-three factorial into High SES and Low SES groups according to Townsend Deprivation Index (Townsend, 1987), an index of poverty (UKB Data-Field 189, acquired at recruitment).

Using data from the current and remitted depressed samples, we conducted an additional test of immunological and metabolic effects associated with anti-depressant medication load. We did this by first extracting from the UKB tabular data for each participant UKB-coded, current and ongoing medications (UKB Data-Field, 20003) and then matching these endorsed medications with the World Health Organization's Anatomical Therapeutic Chemical classification of antidepressant drugs (https://www.whocc.no/atc_ddd_index/?code=N06A). For the Medication Status factor, participants were sorted into Nonmedicated Depressed, One-Medication Depressed, and Two-or-More-Medications Depressed groups.

As an ancillary test of the co-occurrence of innate immune and metabolic dysfunction, we examined in each group separately the rank-order correlation between CRP and MBS by calculating Spearman's rho using the MATLAB function corr. We then calculated 95 % confidence intervals of these correlation estimates using bootstrapping implemented in MATLAB. We observed modest (average rho = 0.33) but consistently significant positive correlations between CRP and MBS across all groups. See Supplementary Fig. 3.

#### 2.3.3 Between-group analysis

**Fig. 2:**
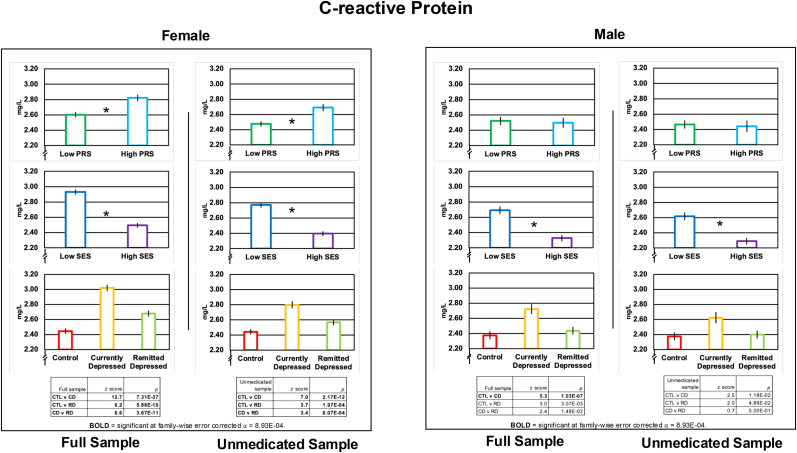
Groupwise mean ( ± standard error) C-reactive protein in females and males as a function of Depression Status (above), Genetic Risk (middle), and Socioeconomic Status (below). CD = Currently Depressed; CTL = Control; PRS = polygenic risk score; RD = Remitted Depressed; SES = socioeconomic status. Significant (*p* < 8.93E-04) effects of Genetic Risk and SES are indicated by an asterisk. The statistical tables below the Depression Status effects shows the results of Wilcoxon Rank Sum tests conducted to further analyze the significant omnibus effect of Depression Status. In the table, rows in **BOLD** = significant at family-wise error corrected ⍺ = 8.93E-04.

**Fig. 3:**
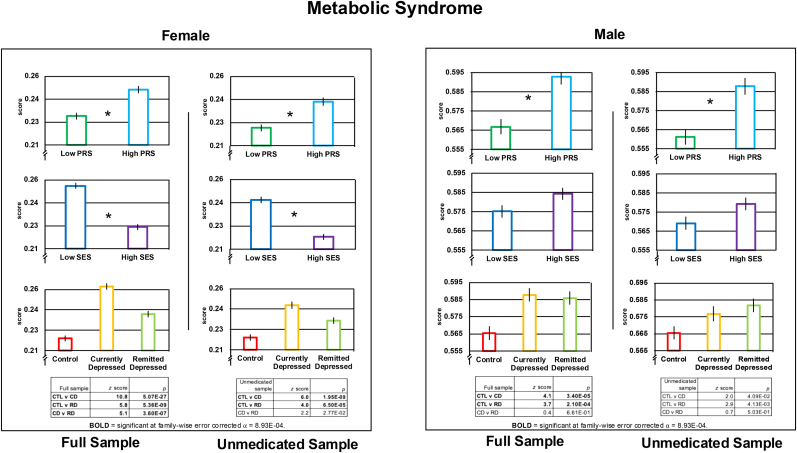
Groupwise mean ( ± standard error) Metabolic Syndrome score in females and males as a function of Depression Status (above), Genetic Risk (middle), and Socioeconomic Status (below). CD = Currently Depressed; CTL = Control; PRS = polygenic risk score; RD = Remitted Depressed; SES = socioeconomic status. Significant (*p* < 8.93E-04) effects of Genetic Risk and SES are indicated by an asterisk. The statistical tables below the Depression Status effects shows the results of Wilcoxon Rank Sum tests conducted to further analyze the significant omnibus effect of Depression Status. In the table, rows in **BOLD** = significant at family-wise error corrected ⍺ = 8.93E-04.

**Fig. 4:**
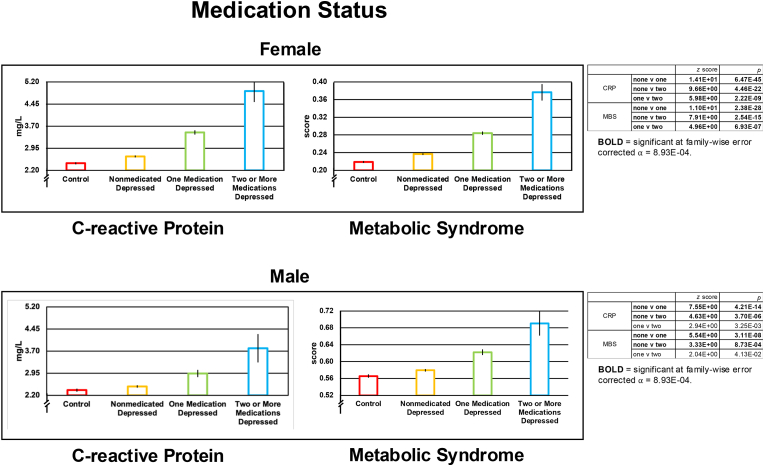
Groupwise mean ( ± standard error) C-reactive protein and Metabolic Syndrome score in females and males as a function of Medication Status. Data from Controls were not included in the statistical analysis but are shown as points of reference. CRP = C-reactive protein; MBS = Metabolic Syndrome score. The statistical table to the right of the Medication Status effects shows the results of Wilcoxon Rank Sum tests conducted to further analyze the significant omnibus effect of Medication Status. In the tables, rows in **BOLD** = significant at family-wise error corrected ⍺ = 8.93E-04.

Neither CRP nor MBS score met the assumption of normality—both *p* < 0.05 for one-sample Kolmogorov-Smirnov tests (implemented with the MATLAB function kstest). Nonetheless, as analysis of variance (ANOVA) is robust to violations of the assumptions of normality (Schmider et al., 2010), we used ANOVA for all omnibus tests (MATLAB function anovan; Type III sum of squares). We conducted: 1) one two-by-three ANOVA with one Genetic Risk fixed factor (lower, “Low,” versus upper, “High,” PRS quartile) and one Depression Status fixed factor (Control versus Currently Depressed versus Remitted Depressed) in both female and male samples; 2) one two-by-three-by-two ANOVA adding an SES fixed factor (less than [“Low”] versus greater than [“High”] the median SES) to the primary model; and 3) a one-by-three ANOVA examining associations between antidepressant Medication Status (Nonmedicated Depressed versus One-Medication Depressed versus Two-or-More Medications Depressed) and the immunological and metabolic variables. To determine the composition of significant effects identified by ANOVA, we conducted follow-up, cell-wise comparison tests. Given concerns about the robustness of the *t-*statistic to violations of the assumption of normality (Canavos, 1988), we used a non-parametric alternative, the Wilcoxon rank sum test (MATLAB function ranksum) for these follow-up contrasts; for convenience, these results are presented under Fig. 2, Fig. 3, Fig. 4 Supplementary Fig. 2as *z* statistics.

## 3 Results

### 3.1 Co-aggregation of genetic risk, depression status, socioeconomic status, and medication status

For descriptive purposes, in the full, non-propensity-selected sample, we examined the coincidence, in terms of frequencies, among the four factors of interest in the study. In both females and males, we saw high rates of co-aggregation across the factors. In High-versus-Low PRS we observed a larger proportion of Low-SES participants, a larger proportion of depression-history positive participants, and a higher rate of psychotropic medication use in the depression-history positive participants. In Low-versus-High SES we saw a higher proportion of depression-history positive participants and more medication use in depression-history positive participants. Finally, in the Current versus Remitted Depressed groups we observed higher rates of medication use. All *p* of χ^2^ tests <4.63E-09. See Table 1 for percentages and inferential statistics.

**Table 1:** Frequency-based co-aggregation in the full, non-propensity matched sample among the factors Genetic Risk, Depression Status, Socioeconomic Status, and Medication Status in Female (lower left) and male (upper right) samples.

	**Genetic Risk**	**Socioeconomic Status**	**Depression Status**	**Medication Status**
**Genetic Risk**		Proportion low-SES greater in high (51.5 %) than low (46.0 %) PRS groups; $\chi^{2}$ = 15.69.	Proportion depression-history-positive greater in high (36.6 %) than low (22.9 %) PRS groups; $\chi^{2}$ = 36.49.	Proportion medicated among depression-history-positive greater in high (16.9 %) than low (12.2 %) PRS groups; $\chi^{2}$ = 9.71.
**Socioeconomic Status**	Proportion low-SES greater in high (51.44 %) than low (46.81 %) PRS groups; $\chi^{2}$ = 14.04.		Proportion depression-history-positive greater in low (31.59 %) than high (28.84 %) SES groups; $\chi^{2}$ = 17.92.	Proportion medicated among depression-history-positive greater in low (20.1 %) than high (17.7 %) SES groups; $\chi^{2}$ = 5.86.
**Depression Status**	Proportion depression-history-positive greater in high (52.8 %) than low (37.7 %) PRS groups; $\chi^{2}$ = 46.05.	Proportion depression-history-positive greater in low (48.26 %) than high (42.16 %) SES groups; $\chi^{2}$ = 41.25.		Proportion medicated greater in current (24.6 %) than remitted (9.0 %) depression groups; $\chi^{2}$ = 32.0.
**Medication Status**	Proportion medicated among depression-history-positive greater in high (21.0 %) than low (16.2 %) PRS groups; $\chi^{2}$ = 12.30.	Proportion medicated among depression-history-positive greater in low (20.1 %) than high (17.7 %) SES groups; $\chi^{2}$ = 6.07.	Proportion medicated greater in current (27.0 %) than remitted (13.5 %) depression groups; $\chi^{2}$ = 34.3.	

Note: All p < 4.63E-09.

### 3.2 Genetic risk-by-depression status-by-socioeconomic status

Addition of an SES factor to the primary analysis (Genetic Risk-by-Depression Status) showed an SES effect and did not change the pattern of results from the primary analysis. We therefore present only the Genetic Risk-by-Depression Status-by-Socioeconomic Status results here. See Supplementary Tables 2 and 3 for ANOVA tables from both models. All effects noted as significant here and hereafter are significant beyond family-wise-error corrected α = 8.93E-04 (family-wise *p* = 0.05 over 56 tests).

#### 3.2.1 CRP

In females, we observed significant main effects of Genetic Risk, Depression Status, and SES but not of the interactions between these factors. CRP levels were significantly higher in High-versus-Low PRS, Low-versus-High SES, and in Currently Depressed versus Control, Currently Depressed versus Remitted Depressed, and in Remitted Depressed versus Control. This same pattern of results was observed after removing data from participants taking psychotropic medications. See Fig. 2 and Supplementary Tables 3 and 4

In males, we saw a significant effect of Depression Status and SES but not Genetic Risk and not the interaction of these factors. The main effect of Depression Status was driven by significantly elevated CRP in the Currently Depressed versus Control groups but this effect was not observed when only unmedicated participants were considered. The effect of SES was due to significantly higher CRP in the Low-versus-High SES group and this effect was observed in both the full and unmedicated samples. See Fig. 2 and Supplementary Tables 3 and 4

#### 3.2.2 MBS

In the female sample, we saw significant effects associated with MBS of Genetic Risk, Depression Status, and SES but not of the interactions between these factors. Just as for CRP, MBS was significantly higher in High-versus-Low PRS, in Low-versus-High SES, and in Currently Depressed versus Control, Currently Depressed versus Remitted Depressed, and in Remitted Depressed versus Control. This same pattern occurred when considering only unmedicated participants with the exception that Currently Depressed and Remitted Depressed groups did not differ significantly once potential effects of psychotropic medication were removed. See Fig. 3 and Supplementary Tables 3 and 4

In the male sample, we saw significant effects associated with Genetic Risk and Depression Status but not SES and not arising from interactions between these factors. The effect of Genetic Risk was due to significantly higher MBS in High-versus-Low PRS, an effect also observed in the unmedicated sample. The effect of Depression Status was driven by significantly higher MBS in both the Currently Depressed and Remitted Depressed groups relative to the Control group, but these effects did not remain when considering only unmediated participants. See Fig. 3 and Supplementary Tables 3 and 4

### 3.3 Medication status

#### 3.3.1 CRP and MBS

In females, we observed similar and significant effects of Medication Status associated with CRP and MBS. The Two-or-More Medications group had significantly higher CRP and MBS than both the Nonmedicated group and the One-Medication group; similarly, the One-Medication group had significantly higher CRP and MBS than the Unmedicated group. We also saw similar significant effects of Medication Status in males on CRP and MBS. The Two-or-More Medications group had significantly elevated CRP and MBS relative both to the Nonmedicated group and the One-Medication group. We did not observe, however, a significant difference in CRP or MBS between the One- and Two-or-More Medications groups. See Fig. 4 and Supplementary Table 5. To see these same effects in Currently Depressed and Remitted Depressed samples separately, see Supplementary Fig. 4.

## 4 Discussion

In this study we examined polygenic risk for depression, depressive-disorder status, and the interaction of these factors in relation to metabolic and immunological variables in women and men. We added to this primary model socioeconomic and psychotropic-medication status as ancillary factors. Importantly, before de-confounding with propensity matching and analysis using the factors of interest, we see natural co-aggregation of High PRS, Low SES, lifetime incidence of depression, and psychotropic medication use. In the analyzed sample, we saw, in women, main effects of Genetic Risk, Depression Status, Socioeconomic Status, but not interactions among them. Specifically, in the higher-versus-lower PRS quartile and in those below-versus-above the median SES we saw elevated indicators of metabolic syndrome and immunological signaling. Additionally, in currently depressed persons relative both to remitted depressed and healthy controls, and in remitted depressed individuals relative to healthy controls, we saw elevated metabolic and immunological disturbance. This pattern remained when excluding from the analysis individuals taking psychotropic medications with the exception that unmedicated current- and unmedicated remitted-depressed groups did not significantly differ with respect to metabolic dysfunction. In men, we observed a different pattern of results where lower-versus-higher socioeconomic status was associated with elevated immunological signaling and higher-versus-lower polygenic risk for depression was associated with elevated metabolic dysfunction. Finally, in both men and women, we saw that increasing psychotropic medication use was associated with elevated indicators of immune and metabolic disturbance.

With its combination of genetic- and environmental-risk factors and disease-status factors, the present investigation can address the epidemiological significance of immunological and metabolic functioning in depression, where different possible patterns in the data can constitute distinct epidemiological signatures. For example, if we had seen that indicators of immune and metabolic dysfunction were only aberrant in current depression and were not associated with genetic- or environmental-risk then we might infer that these indicators co-emerge with or potentially form some of the molecular basis of depressive illness. In contrast, if these indicators were elevated in association with genetic- and environmental-risk but not with state-level depressive illness then we might conclude that they comprise part of a risk-basis for depression that interacts with other factors in the pathophysiology of the disease state. In reaching epidemiological conclusions using immune and metabolic data, however, it is important to acknowledge that temporality in the immune-metabolic domain is highly variable at inter- and intra-individual levels (Metcalf et al., 2025) and is unlikely to map perfectly onto trajectories of risk, occurrence, and relapse in major depression.

In women, we observed that markers of immunological and metabolic dysfunction were elevated in both genetic- and environmental-risk for depression, in state depression, and in remitted depression (see Fig. 2, Fig. 4). In other words, these markers are elevated in relation to more distal genetic- and environmental-risk factors but also, more proximally, in the disease state itself. Further, and importantly, the genetic- and environmental-risk and disease-status factors did not interact significantly. One interpretation of this pattern of data from an epidemiological perspective is that immunological and metabolic factors each have a summation effect in the pathophysiology of depression such that the higher the dysfunctional load, the greater risk of depression. In this context our findings and those from earlier investigations of genetic and disease-status factors in the immunology of depression (Pitharouli et al., 2021; Sewell et al., 2021) contribute to a body of knowledge for more accurately identifying and addressing depressive-subtype-specific needs upon presentation in the clinic. Our findings indicate, for example, that women presenting with depressive symptoms and who suffer from socioeconomic deprivation and have elevated genetic risk for MDD (which can now be assessed relatively quickly and easily) are statistically more likely to suffer from immune-metabolic disturbance and can be treated accordingly.

Our finding of increased metabolic and immunological dysfunction with increasing use of psychotropic medication presents a quandary. Given that psychotropic medications are effective in treating major depression (Yuan et al., 2020) and that metabolic (Pan et al., 2012b) and immunological (Jeon and Kim, 2017) dysfunction are putatively causal factors in this disorder, then how can it be that psychotropic medication load is associated with heightened metabolic and immunological dysfunction? It is possible that these correlational findings arise because more severe depression is associated with more metabolic and immunological dysregulation and that this results in heightened treatment seeking. However, anti-depressant use is associated with *subsequent* weight gain (Gafoor et al., 2018; Alonso et al., 2019). Assuming that immune and metabolic factors are important in the pathophysiology of depression (Pan et al., 2012b; Jeon and Kim, 2017), weight gain occurring subsequent to psychotropic intervention challenges the idea that the presumably more metabolically challenged treatment seekers in the above scenario would benefit from antidepressants. This position is supported, in part, by findings that overweight and obese individuals are less likely to benefit from antidepressant therapy (Woo et al., 2016). Another possibility is that, in causing weight gain, antidepressants selectively treat melancholic and anxious depressed phenotypes (weight-loss and insomnia), normalizing these symptoms as they transition toward an atypical phenotype (weight-gain, and fatigue/hypersomnia). Studies reporting that melancholic and atypical subtypes of depression show similar responses to pharmacotherapy (Cuijpers et al., 2017), however, challenge this assertion.

The issues faced by the more elegant hypotheses concerning the medication effects considered here invite more complex formulations. To the extent that the pathophysiological burden of metabolic and immune disturbance translates to the domain of negative affective and bodily signals, it is possible that antidepressants change this and render it inert. There is strong evidence that antidepressant therapy results in affective blunting in a majority of cases (Jawad et al., 2023; Read and Williams, 2018). This blunting, itself, could be reflected in the consistently observed reduction in baseline insula activity in response to serotonergic antidepressants (Chau et al., 2017). It is possible, then, that medicated, affectively blunted persons experience a general reduction in depressotypic, negative affective and bodily signals and remain on a course to continued immune-metabolic disturbance in the relative absence of negative feedback. Of course, it is also possible that unassessed variables embedded in the medication effects can help better explain the enigmatic aspects of our findings. For example, there is significant variability in terms of effects on immune and metabolic processes among the broad array of medications considered as antidepressants here (Gafoor et al., 2018; Alonso et al., 2019; Więdłocha et al., 2018). Accounting for this variability in future research might provide additional clarity in this regard.

Our methodological choice to propensity-match female and male samples separately precludes direct comparison between them. Nonetheless, and consistent with our hypotheses, we observed more consistent associations among genetic, socio-economic, disease-status, and immune-metabolic factors for females than for males. There are at least two accounts of this discrepancy. The first is that, as we hypothesize based on prior work, genetic, socio-economic, disease-status, and immune-metabolic factors converge to a greater degree in women than men, but for reasons that remain unaddressed. The second is that our design is more sensitive for identifying effects in women than in men since the female sample is twice the size of the male sample. Some similarities in the patterns of results between the female and male samples suggest that this is partially, but not completely, the case.

There are several important limitations to the present study. First, while the present results contribute to a growing literature indicating that inflammatory and metabolic dysfunction are important in major depression, we cannot attribute causal influence to any of the factors investigated here. We employ propensity matching in the present investigation to address several prospective confounders, but other unaddressed confounds and pathogenic variables embedded within the factors of interest could account for our findings. Moreover, in yielding more controlled and homogenous sub-samples for comparison, propensity matching could inadvertently result in selection bias. Additionally, the minimum-age criterion for inclusion in the UK Biobank is 40 years which results in sample ages (mean ≅ 55 years in the present study) that are relatively high for investigations of MDD, in which incidence begins to climb in early adolescence (Lieb et al., 2002). Considering the immunological (Brüünsgaard and Pedersen, 2003) and metabolic (Toth and Tchernof, 2000) changes that occur with advancing age, it is possible that the immune-metabolic effects reported here emerge with age and are, therefore, specific to higher-age cohorts. Further, while several highly significant effects are reported here, we hasten to point out that these effects still explain only small proportions of the variance in the data and, therefore, do not constitute comprehensive accounts of immune and metabolic function. In this same context, it is important to consider that a large variety of innate and adaptive immunological factors are altered in depression (Osimo et al., 2020). Given our focus on genetic, state, and trait factors in immune-metabolic functioning, we have greatly simplified the immunologic profile of depression in considering only CRP and do not assume generalizability beyond the innate-immune domain. Further, our operationalization of metabolic syndrome, MBS, lacks information about fasting glucose levels, a cardinal criterion for metabolic syndrome, which potentially limits the validity of our measure. Moreover, the present investigation would have benefitted from additional characterization of the depressed samples with respect to the atypical-melancholic distinction; nonetheless, we avoided this to maintain a tractable number of dimensions in the analysis. Importantly, the way we operationalize lifetime history of depression as well as current depression would benefit from additional analysis in terms both of the sensitivity and specificity of these definitions. Finally, the genetic effects we report are based on polygenic scores derived from meta-analysis of results from samples of European ancestry. It is possible, then, that our genetic findings would not replicate in non-European samples. This limitation should be heeded particularly considering any epidemiological or therapeutic implications of the findings we present (Martin et al., 2019).

In this investigation, we found in women that markers of immune and metabolic dysfunction track in intuitive ways with genetic and environmental risk for depression as well as state and trait aspects of this disorder. The question of how these pathophysiological processes are elevated with increasing pharmacological intervention remains unanswered. We found, further, discrepant findings between female and male samples where only socioeconomic status and genetic risk tracked, respectively, with immunological and metabolic markers in males. This echoes previous findings that, in depression, pathophysiological processes in women and men are at least partially independent, potentially meriting independent lines of scientific inquiry.

## CRediT authorship contribution statement

**David M. Howard:** Conceptualization, Data curation, Formal analysis, Funding acquisition, Investigation, Methodology, Project administration, Resources, Software, Writing – original draft, Writing – review & editing. **Lachlan Gilchrist:** Formal analysis, Methodology, Software, Writing – review & editing. **Petroula Proitsi:** Formal analysis, Methodology, Resources, Software, Writing – review & editing. **Elisabeth R. Paul:** Conceptualization, Investigation, Methodology. **Markus Heilig:** Conceptualization, Data curation, Funding acquisition. **Lars Östman:** Conceptualization, Investigation, Methodology. **Robin Kämpe:** Conceptualization, Formal analysis, Investigation, Methodology. **J. Paul Hamilton:** Conceptualization, Data curation, Formal analysis, Funding acquisition, Investigation, Methodology, Project administration, Resources, Software, Supervision, Writing – original draft, Writing – review & editing.

## Declaration of competing interest

Dr. Howard, Dr. Paul, Dr. Östman, Mr. Kämpe, and Dr. Hamilton report no financial relationships with commercial interests. Dr. Heilig has received consulting fees, research support or other compensation from Indivior, Camurus, BrainsWay, Aelis Farma, and Janssen Pharmaceuticals, none of which are relevant to the presented work.
