# Supplementary material for: Immune and metabolic disturbance as a function of genetic risk and phase of illness in major depression
Source: Brain Behav Immun Health. 2025 Nov 27;50:101144. doi: 10.1016/j.bbih.2025.101144 (PMC12718154; doi:10.1016/j.bbih.2025.101144)
Supplement: Multimedia component 1 [file mmc1.docx]

Methods

*Data source*

The UKB is a prospective cohort study of over a half-million individuals aged 40–69 years at first assessment. Initial UKB enrollment took place between 2006 and 2010 in 22 assessment centers across the United Kingdom.^1^ The UKB has a comprehensive array of health-related biological and behavioral data including genome-wide genotyping, blood biochemistry, clinical exam results, and self-reported health indices. All UKB participants provided informed consent. Ethical approval to the UKB is provided by the UK Biobank research ethics committee as well as the Human Tissue Authority research tissue bank. Adherence to the Ethics and Governance Framework by the UKB is overseen by an independent Ethics and Governance Council. The current study was approved by the UKB (study ID 49037).

*Dependent measures*

Supplementary Figure 1. In female and male samples, the number of participants per the number of measures used in calculating the Metabolic Syndrome score.

*Data analysis*

**Covariates / confounder control.**

****Supplementary Figure 2. Groupwise mean age (+/- standard error) and associated main effects and interactions before (A) and after (B) propensity matching of female samples. Dx = diagnosis = Depression Status; PRS = polygenic risk score = Genetic Risk.

**Ancillary analyses.**

Supplementary Figure 3. Groupwise Spearman correlations (+/- 95% confidence interval) between C-reactive Protein (CRP) and Metabolic Syndrome score (MBS) in Low- and High-Socioeconomic Status (SES), female and male samples. PRS = polygenic risk score.

Results

*Genetic Risk-by-Depression Status*


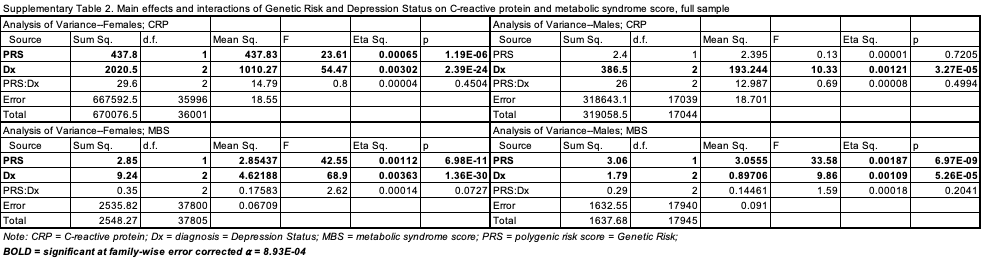


*Genetic Risk-by-Depression Status-by-Socioeconomic Status, full testing sample*


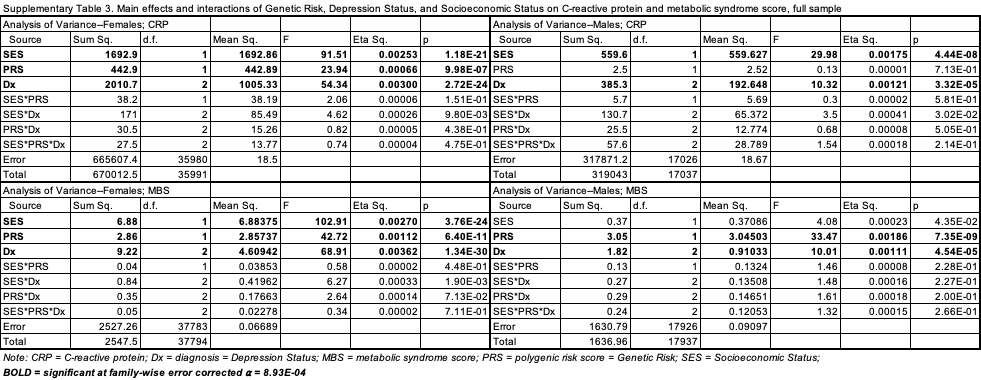


*Genetic Risk-by-Depression Status-by-Socioeconomic Status, unmedicated sample*


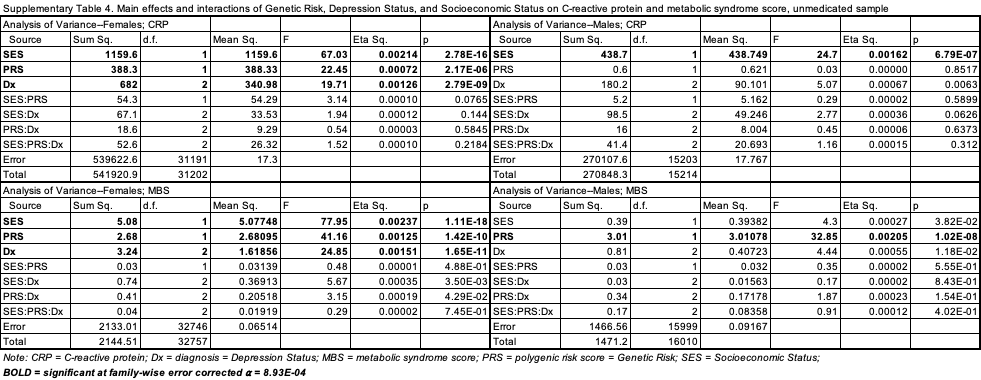


*Medication Status*


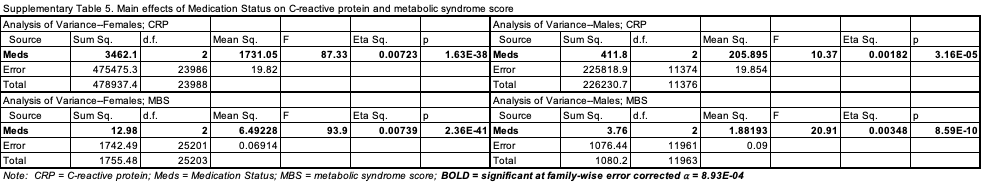

Supplementary Figure 4. Groupwise mean (+/- standard error) C-reactive protein and Metabolic Syndrome score in females and males as a function of Medication Status. This figure complements Figure 4 in the main text in presenting separately data from Remitted Depressed and Currently Depressed groups. Data from Controls were not included in the statistical analysis but are shown as points of reference. CRP = C-reactive protein; MBS = Metabolic Syndrome score.

1 Sudlow, C. *et al.* UK Biobank: An Open Access Resource for Identifying the Causes of a Wide Range of Complex Diseases of Middle and Old Age. *Plos Medicine* **12** (2015). <https://doi.org:10.1371/journal.pmed.1001779>
